# Evaluation of *Candida* spp. and Other Fungi in Feces from Dogs with Naturally Occurring Diabetes Mellitus

**DOI:** 10.3390/vetsci9100567

**Published:** 2022-10-16

**Authors:** Jared A. Jaffey, Ogi Okwumabua, Thomas K. Graves, Layla Al-Nakkash, Ross Monasky, Alec Wilson, Shankar Thangamani

**Affiliations:** 1 Department of Specialty Medicine, College of Veterinary Medicine, Midwestern University, Glendale, AZ 85308, USA; 2 Department of Pathology, College of Veterinary Medicine, Midwestern University, Glendale, AZ 85308, USA; 3 Department of Physiology, College of Graduate Studies, Midwestern University, Glendale, AZ 85308, USA; 4 Department of Comparative Pathobiology, College of Veterinary Medicine, Purdue University, West Lafayette, IN 47906, USA; 5 Purdue Institute for Immunology, Inflammation and Infectious Diseases (PI4D), West Lafayette, IN 47906, USA

**Keywords:** mycobiome, microbiome, endocrine, hyperglycemia, glycemic control, fructosamine

## Abstract

**Simple Summary:**

Diabetes mellitus is a common endocrine disorder in dogs that is similar to type 1 diabetes mellitus (T1DM) in humans. *Candida* spp. is a common non-pathogenic fungi that is identified more commonly and in higher amounts in humans with T1DM, including the gastrointestinal tract. This change to the distribution of microorganisms that inhabit the intestine has potential to affect glycemic control and even spread to other organs and cause severe illness. There are no studies that have investigated whether diabetic dogs, like humans, have alterations to the intestinal mycobiome. Therefore, our study sought to determine whether differences exist in the types of fungi cultured from feces in diabetic dogs and non-diabetic healthy control dogs. In addition, we wanted to find out if there were variables associated with fungi colonization. Diabetic dogs had more quantitative fungal growth than controls and females were more likely to yield growth than males. Diabetic dogs were also more likely to have *Candida* spp. colonized from feces. Glycemic control was also seemingly associated with growth of *Candida* spp. in diabetic dogs. Our results indicate that the intestinal mycobiome is altered in diabetic dogs with increased prevalence of *Candida* spp. and quantitative growth of fungi.

**Abstract:**

Diabetes mellitus is a common endocrinopathy in dogs and in most cases is analogous to type 1 diabetes mellitus (T1DM) in humans. *Candida* spp. is a common commensal fungi with higher prevalence and magnitude of growth in humans with T1DM. There is currently no published information about the fungal microbiome in diabetic dogs. Therefore, the objectives of this study were to (i) determine whether diabetic dogs were more likely to have *Candida* spp. or other types of fungi from feces compared to non-diabetic controls, and (ii) identify variables associated with fungi colonization. Fourteen diabetic dogs and 14 age, sex, and breed matched non-diabetic healthy control dogs were included in this prospective case–control study. Matrix assisted laser desorption/ionization time-of-flight mass spectrometry (MALDI-TOF-MS) was used for fungal identification. Diabetic dogs had greater quantitative fungal growth compared to controls (*p* = 0.004). Moreover, female dogs were more likely to have fungi colonization than males (*p* = 0.02). All instances of *Candida* spp. and *Aspergillus* spp. colonization were exclusively identified in diabetic dogs. Serum fructosamine concentration was higher in diabetic dogs with fecal colonization of *Candida* spp. compared to diabetic dogs without growth (*p* = 0.03). Our results indicate that the fungal microbiome in feces is altered in diabetic dogs, which seem to favor an increased prevalence of *Candida* spp. and higher quantitative fungal growth. Moreover, female sex and glycemic control could affect the intestinal mycobiome.

## 1. Introduction

Diabetes mellitus is a common endocrinopathy in middle to older aged dogs. The estimated prevalence in pet populations varies with geographic region, ranging between 0.2–1.3%, and is even higher in predisposed breeds [1,2,3,4,5,6,7,8,9]. A recent report estimated that approximately 165,000 dogs in the United States have diabetes mellitus, assuming a pet population of 70 million dogs [10]. The most common clinically recognized form of diabetes mellitus in dogs is analogous to type 1 diabetes mellitus (T1DM) in humans [11]. 

*Candida* species are common fungi generally considered commensal organisms present in the intestinal tract of dogs and humans [12,13,14,15]. However, alterations in intestinal microbiota and host immunologic defenses can lead to tissue invasion resulting in substantial local and systemic disease [15,16,17,18,19,20]. Type 1 diabetes mellitus predisposes humans to intestinal and urinary *Candida* spp. overgrowth as well as systemic candidiasis [21,22,23,24,25,26,27]. Various mechanisms are purported to be responsible for increased colonization and virulence of *Candida* spp. in this environment, such as increased yeast adhesion to epithelium, impaired candidacidal activity of neutrophils, altered yeast hemolytic/esterase enzymatic activity, and intestinal bacterial dysbiosis [26,28,29,30,31,32]. Currently, there is a void in the veterinary literature regarding the influence of diabetes mellitus on the fungal microbiome in dogs.

Improving our understanding of the fungal microbiome in dogs with naturally acquired diabetes mellitus could provide a foundation with which future studies investigate the relationship between the fungal microbiome and clinical course of diabetes mellitus in dogs. Therefore, this study had two objectives: (i) to determine whether dogs with naturally occurring diabetes mellitus (NODM) were more likely to have *Candida* spp. and other types of fungi colonized from feces compared to non-diabetic control dogs, and (ii) to identify variables associated with fungi colonization. We hypothesized that dogs with NODM would be more likely to have fungi colonized (including *Candida* spp.) from feces and more than one variable would be associated with its presence.

## 2. Materials and Methods

### 2.1. Criteria for Selection of Cases and Study Design

Client-owned dogs with NODM treated with ≥0.25 units/kg of insulin administered once every 12 h presented to the Companion Animal Clinic at Midwestern University College of Veterinary Medicine (MWU-CVM) were eligible for inclusion. Diabetic dogs were classified as having clinically well-regulated diabetes mellitus if the following conditions were met: no polyuria, polydipsia, polyphagia, and there had been no insulin dose adjustments within 4 weeks of enrollment. Dogs were excluded if they were obese (i.e., body condition score of ≥6/9), received vaccination or medications expected to alter the intestinal microbiota (e.g., antibiotics or antifungals) within 1 month of enrollment. Monthly parasiticides were permitted. Dogs with relevant comorbidities or concurrent illness within 60 days of enrollment were also excluded. Determination of clinical relevance of comorbidity was made by a board-certified small animal internist. A second population of healthy, age (i.e., ±2 years of NODM counterpart), breed, and sex-matched non-diabetic healthy controls were enrolled. Control dogs were included after a review of clinical history, physical examination, complete blood count, and serum chemistry by a single investigator. Control dogs were enrolled in the study if they met the following requirements: non-obese, no illnesses within 6 months of enrollment, had not received a vaccination, or medications expected to alter intestinal microbiota within 1 month of enrollment. Monthly parasiticides were permitted. All dogs were included in the study after obtaining informed owner consent. This study was conducted in accordance with guidelines for clinical studies and approved by the MWU Animal Care and Use Committee (protocol: #2944; approval date: 14 June 2019).

### 2.2. Data and Sample Collection

Medical records were reviewed for each dog enrolled. The age, sex, weight, and breed were recorded for each. Other relevant details were recorded when indicated: maintenance diet information, insulin type and dosage, and miscellaneous medications. All hematology and biochemical parameters were measured at a commercial laboratory (Antech Diagnostics, Fountain Valley, CA, USA). Fecal samples were collected after natural voidance. Fecal samples were stored at 4 °C for ≤24 h before culture and subsequently stored frozen at −80 °C until DNA extraction.

### 2.3. Fungal Culture, Isolation, Quantification Methods

Fungal burden was assessed based on previously established protocols in animal models of infection [33,34]. Approximately 50 mg of each fecal sample was weighed and homogenized in 1 mL of sterile phosphate-buffered solution (PBS). Samples were weighed so the total mass of each sample used in fungal enumeration was known and could be used to calculate fungal burden following plating. Next, 100 µL of fecal homogenate was then serially diluted in sterile PBS to a final dilution of 10^−7^ for a total of 8 dilutions (a neat sample, 10^−1^, 10^−2^, 10^−3^, 10^−4^, 10^−5^, 10^−6^, and 10^−7^). These dilutions of fecal homogenate were then plated on yeast peptone-dextrose agar plates containing kanamycin, ampicillin, and streptomycin, each at a concentration of 50 µg/mL. Fecal homogenization, serial dilution, and plating was performed in triplicate to have a representative evaluation of each sample. Plates were incubated at 30 °C for 48 h to allow for fungal colony growth [35,36]. Fungal burden was then calculated by counting colony forming units (CFU) at the lowest dilution where colonies were distinct in all replicates, multiplying this count by the dilution factor, and then dividing by the total mass of feces processed for the sample, generating a measure of CFU per gram of feces. Unique fungal colonies were preserved by homogenizing colonies in yeast extract-peptone dextrose (YBD) broth containing 15% glycerol and freezing at −80 °C.

### 2.4. Identification Methods

Representative individual colonies recovered from quantification plating were identified using matrix assisted laser desorption/ionization time-of-flight mass spectrometry (MALDI-TOF-MS) analysis following the manufactures protocol. Both non-filamentous and filamentous fungal species were recovered during initial plating, and samples were processed to identify fungal species using MALDI-TOF as per manufacturer instructions (Bruker Daltonics, Billerica, MA, USA). In brief, fungal isolates were inoculated in Sabourand liquid broth (BD Life Sciences, Huntsville, AL, USA) and incubated for 24 h at 26 °C for growth. Fungal growth was collected and washed two times with HPLC-grade water (MilliporeSigma, Burlington, MA, USA) and once with molecular grade ethanol (ThermoFisher, Waltham, MA, USA). Washes consisted of re-suspending the fungal pellet in the wash medium, followed by a 2-min centrifugation at room temperature at 13,000 rcf. Following the ethanol wash, the samples were dried until all residual ethanol evaporated. The dried pellet was then re-suspended in 70% formic acid and acetonitrile. The sample was then centrifuged and the supernatant was spotted on a MALDI-TOF-MS target plate, allowed to air dry, and then covered with a 1 µL HCCA matrix (Bruker Daltonics Billerica, MA, USA) and analyzed on a MALDI-TOF-MS biotyper (Bruker Daltonics, Billerica, MA, USA).

### 2.5. Statistical Analysis

Statistical analysis was performed by commercial software (SigmaPlot, Systat Software, version 13). A Shapiro-Wilk test was used to assess normality. Categorical data were presented as proportions. Non-normally distributed continuous data were described as median and interquartile range (IQR). Normally distributed continuous data were presented as mean and standard deviation (SD). Student’s t-test was used for two group comparisons of normally distributed continuous variables, and Mann-Whitney rank sum test for non-normally distributed continuous variables. Fisher’s exact test was used for categorical associations. A *p*-value of <0.05 was considered significant. The data presented in this study are openly available. Canine Diabetes Mellitus *Candida* spp. Available online: http://www.kaggle.com/jaredjaffey/canine-diabetes-mellitus-candida-spp?rvi=1 (accessed on 10 October 2022).

## 3. Results

### 3.1. Animal Population

A total of 28 dogs were eligible for inclusion in this prospective case–control study. There were 14 NODM dogs and 14 non-diabetic controls. No dogs were excluded. Descriptive data are summarized in Table 1. Diabetic dogs were maintained on either neutral protamine Hagedorn (NPH) (50%, 7/14) or porcine lente (50%, 7/14) insulin. The median dose of insulin administered once every 12 h to NODM dogs was 0.7 units/kg (IQR, 0.5; range, 0.3–1.1). Seven diabetic dogs were clinicall well-regulated and the remaining 7 dogs were not. Dogs were fed a variety of commercially available pet foods. Details regarding diet information can be found in Supplemental Material. 

Diabetic dogs had higher serum glucose (*p* = 0.01), fructosamine (*p* < 0.001), triglyceride (*p* = 0.04), and cholesterol (*p* < 0.001) concentration and were more likely to have glucosuria (*p* < 0.001) than non-diabetic controls (Table 2).

### 3.2. Fungi Characterization and Distribution

**Figure 1:**
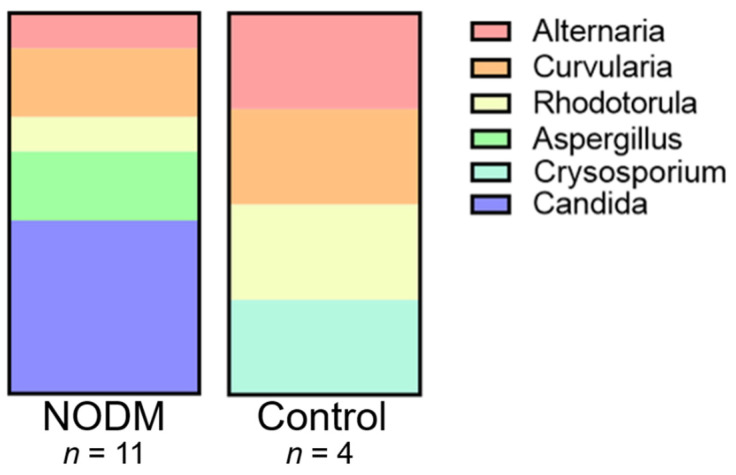
Relative distribution of fungal genera colonized from fecal cultures in dogs with naturally occurring diabetes mellitus and non-diabetic healthy controls.

Fungal colonies were cultured from fecal samples in 57% (16/28) of dogs (NODM, *n* = 10; non-diabetic controls, *n* = 6). Five dogs (NODM, *n* = 2; non-diabetic controls, *n* = 3) with fungal growth from fecal cultures had mixed multiple types of colonies that could not be identified by the MALDI system. Most of the remaining dogs in both groups with fungal growth had a single individual fungal colony identified from fecal cultures. Two diabetic dogs with fungal growth had multiple distinct colonies isolated. Specifically, one diabetic dog each had two and three colonies isolated, respectively. Likewise, one control dog had two distinctive fungal colonies isolated. In total, the NODM group had 11 colonies successfully identified and the non-diabetic control group had 4 identified colonies (Table 3). From these 15 positive identifications, 9 distinct species across 6 genera were found. Half of the genera identified were shared between the NODM and non-diabetic control groups, including *Alternaria*, *Curvularia*, and *Rhodotorula*, however all instances of *Candida* and *Aspergillus* colonization were exclusively identified in the NODM group (Figure 1). *Candida* spp. accounted for 45% (5/11) of fungal species identified in dogs with NODM (Table 3).

### 3.3. Risk Factors for Fungi Colonization

**Figure 2:**
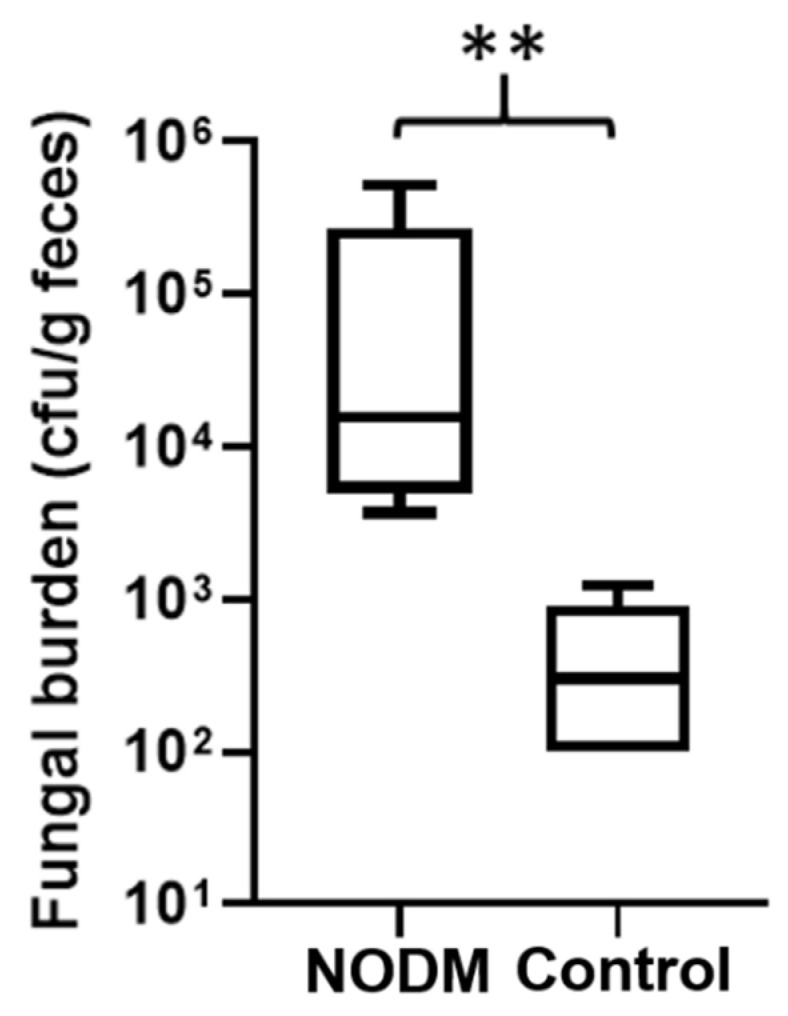
Box and whisker plot comparing quantitative fungi growth from fecal cultures in dogs with naturally occurring diabetes mellitus (*n* = 5) and non-diabetic healthy controls (*n* = 6). The top and bottom of the boxes represent the 75th and 25th quartiles, respectively with the black horizontal line representing the median. The whiskers represent the range of data. ** *p* = 0.004.

There was no difference (*p* = 0.25) in the proportion of NODM dogs (71%, 10/14) and non-diabetic controls (43%, 6/14) that had fungal colonies isolated from fecal cultures. In addition, there were no differences in age, weight, or selected serum biochemical parameters (e.g., glucose, fructosamine, triglycerides, cholesterol) between dogs with or without fungal colonization (Table 4). A greater proportion of female dogs (83%, 10/12) had fungal colonization of fecal cultures than male dogs (38%, 6/16; *p* = 0.02) (Table 4). Quantitative assessment of fungi growth from fecal cultures was performed in 69% (11/16) of dogs (NODM, *n* = 5; non-diabetic controls, *n* = 6) that had fungal colonies isolated. Due to filamentous overgrowth, an accurate quantification of colony forming units was not possible in 31% (5/16) of dogs. These filamentous fungi were isolated and processed for identification, however, were not included in the assessment of fungal burden. *Candida* spp. was the only fungal strain isolated from the five diabetic dogs in this assessment. Three of the six non-diabetic controls used in the quantitative fungal burden analysis had colonies identified via MALDI-TOF biotyping. Two dogs each had a single type of fungi (*Rhodotorula mucilaginosa* and *Chrysosporoum shanxiense*) and one dog had two strains (*Alternaria alternate* and *Curvularia lunata*). Of the samples where an accurate colony count was possible, diabetic dogs had significantly greater quantitative fungal growth from fecal cultures than non-diabetic controls (*p* = 0.004) (Figure 2). 

Dogs with NODM (36%, 5/14) were significantly more likely to have *Candida* spp. colonies isolated from fecal cultures than controls (0%, 0/14, *p* = 0.04). When considering all dogs in our sample population, those with *Candida* spp. fecal colonization had higher serum glucose (*p* = 0.01), fructosamine (0.002), and cholesterol (*p* = 0.01) concentration than dogs without colonization (Table 5). The next aspect of the investigation was to determine if there were risk factors related to *Candida* spp. isolation from fecal cultures in dogs with NODM only. Diabetic dogs with fecal *Candida* spp. colonization had higher serum fructosamine concentration than diabetic dogs without *Candida* spp. growth (*p* = 0.03) (Table 6).

## 4. Discussion

This exploratory study investigated the prevalence and risk factors for colonization of *Candida* spp. and other types of fungi from feces in dogs with NODM. In our investigation, we found that diabetic dogs were more likely to have *Candida* spp. isolated from feces than non-diabetic controls. In addition, serum fructosamine concentration was higher in diabetic dogs with fecal *Candida* spp. colonization than diabetic dogs without growth. While there was no difference in the likelihood of fungi isolation in feces between diabetics and non-diabetic controls, irrespective of fungal strain, diabetic dogs had higher quantitative fungal growth. A higher proportion of female dogs had fungi colonization from feces than males.

Diabetic dogs were significantly more likely to have *Candida* spp. isolated from feces than non-diabetic controls. While intestinal colonization by *Candida* spp. is frequently detected in healthy immunocompetent humans, the frequency and magnitude of quantitative growth is significantly greater in patients with T1DM [23,25,26,37,38]. Comparable to humans, *Candida* spp. is a common component of the intestinal mycobiome in dogs; however, there have been no studies that have investigated it in dogs with diabetes mellitus [14,39,40]. Overall, studies evaluating the interplay between intestinal fungal organisms that comprise the mycobiome and diseases are scarce in dogs [41]. Several mechanisms are surmised to be responsible for intestinal growth of *Candida* spp. in humans with diabetes mellitus such as increased yeast adhesion to epithelium, impaired candidacidal activity of neutrophils, and altered yeast hemolytic/esterase enzymatic activity [26,28,29,30,31,32]. Alterations to the intestinal bacterial microbiome is also thought to increase risk for increased *Candida* spp. colonization in humans with T1DM [26]. *Bifidobacterium*, *Bacteroides*, and *Lactobacillus* species produce short-chain fatty acids and antimicrobial compounds that have inhibitory effects on colonization of fungi. Soyucen et al. found that patients with T1DM had higher *C. albicans* colonization with concomitantly decreased colonization of *Bacteroides* spp. and *Bifidobacterium* spp. compared to non-diabetic healthy controls. Additional research is needed to better understand the pathomechanistic underpinnings of increased intestinal colonization of *Candida* spp. and its clinical effects in diabetic dogs.

Serum fructosamine concentration was higher in diabetic dogs with fecal colonization of *Candida* spp. compared to diabetic dogs without growth. These results support the possibility that intestinal *Candida* spp. load in diabetic dogs could be associated with glycemic control. Serum fructosamine are glycated proteins in blood that are routinely used to monitor glycemic control in diabetic dogs, as they generally represent the mean blood glucose concentration in the preceding 2 to 3 weeks [42,43]. There are limited studies that have investigated the association between glycemic control and colonization of *Candida* spp. in feces or rectum from humans and they have yielded conflicting results [23,25]. In contrast, there is more convincing evidence that support an association between glucose regulation and colonization of *Candida* spp. in the oral cavity and vulvo-vaginal region in diabetic patients [44,45,46,47]. Postulated reasons for this association include magnified glucose mediated immunosuppression and increased pathogenicity of *Candida* spp. (via hydrolytic enzymes) [48]. Another potential mechanism is that during hyperglycemic periods, glycosylation production with proteins accumulate in tissues and buccal epithelial cells, which could increase the number of available receptors for *Candida* spp. [48]. Longitudinal studies with larger sample populations are needed to evaluate if glycemic control is associated with intestinal colonization of *Candida* spp. in diabetic dogs.

Diabetic dogs had significantly higher quantitative growth of fungi from feces than non-diabetic controls. The most likely explanation for this result is that *Candida* spp. was the only isolated fungal strain from diabetic dogs with quantifiable fungal burden available for this comparison. Humans with diabetes mellitus have a significantly higher amount of *Candida* spp. in feces compared to non-diabetic controls [25]. Therefore, our assessment of fungal burden could have been skewed by the composition of fungal strains within the groups. The finding that female dogs were more likely to have fungi isolated from fecal cultures than males was unexpected. Recent research in humans and rodents have shown sex differences in gut microbiota composition [49,50]. However, any sexual dimorphism should have been neutralized because most dogs (93%, 26/28) included in this study were altered (i.e., gonadectomized). There were two intact-female dogs, but only one had fungi colonized from feces. Future studies are needed to fully explore the effect of sex and altered sex status on the intestinal flora in dogs.

Our study had limitations that require further elucidation. We analyzed fungal growth in feces rather than various sites within the intestine. This approach was more practical, non- invasive, and has been commonly employed in humans and dogs to assess intestinal fungi burden [14,25,26,38]. While the use of feces as our sample type for analysis had its clear advantages, it is possible results would have differed had samples been obtained from various sites in the intestine. The incidence of intestinal *Candida* spp. colonization varies in humans depending on the site sampled in the intestinal tract, recovery technique, and transport times [51]. In addition, one study observed a higher prevalence of yeast in the lumen of the jejunum (27%) compared to feces (5%) in dogs [52]. Another limitation is that we utilized culture-based methods to examine the intestinal microbiota, which is not as sensitive as molecular-based methods such as quantitative PCR targeting rRNA or real-time PCR. This could have affected the identification of some fungi, especially those with low abundance. Dogs were excluded if medications expected to alter intestinal microbiota were administered within 1 month of enrollment. The administration of some medications such as antibiotics can affect the intestinal microbiome for months. Therefore, if a dog received one of these medications longer than 1 month before enrollment it might have affected the microbiome. This exploratory study utilized a 1:1 case-control ratio. The use of more controls (e.g., 1:2 or 1:3) could have increased the power of statistical analyses. Lastly, this study evaluated fungal growth in samples obtained on a single occasion. Diabetes mellitus is a dynamic disease and thus, alterations in the microbiota over time are possible, especially in dogs with changes in glycemic control over time.

## 5. Conclusions

In conclusion, the current study provides information about the fungal microbiome present in diabetic and non-diabetic healthy control dogs. Diabetic dogs were more likely to have *Candida* spp. colonized and had higher quantitative fungi growth in feces compared to non-diabetic controls. Serum fructosamine concentration was higher in diabetic dogs with *Candida* spp. isolation compared to diabetic dogs without, which suggests that glycemic control could affect the fungal microbiome. These results provide a foundation for future studies evaluating the fungal microbiome in dogs with NODM and other diseases.

## Figures and Tables

**Table 1:** Baseline descriptive characteristics in dogs with naturally occurring diabetes mellitus and non-diabetic healthy controls.

Variable	NODM	Control	*p*-Value
Number of dogs	14	14	
Age (years) ^a^	9.4 (2.2)	9.5 (2.2)	0.94 ^c^
Weight (kgs) ^b^	8.8 (19.2)	9.8 (21.8)	0.58 ^d^
BCS ^b^	5 (1)	5 (1)	0.71 ^d^
Sex (MN, MI, FS, FI)	8, 0, 5, 1	8, 0, 5, 1	1.0 ^e^
Breeds	Rottweiler, Pomeranian, Labrador retriever, Chihuahua, Havanese, Australian cattle dog, Bichon frise, Miniature pinscher, Yorkshire terrier, Poodle-mix, Labrador retriever-mix, Maltese-mix, Miniature pinscher-mix, Australian shepherd-mix		

BCS, body condition score; kgs, kilograms; NODM, naturally occurring diabetes mellitus; MN, male-neutered; MI, male-intact; FS, female-spayed; FI, female-intact. ^a^ Data presented as mean (standard deviation), ^b^ Data presented as median (interquartile range), ^c^ Two-tailed Student’s *t*-test, ^d^ Mann-Whitney rank sum test, ^e^ Fisher’s exact test.

**Table 2:** Clinicopathologic features of dogs with naturally occurring diabetes mellitus and non-diabetic healthy controls. Data presented as median (interquartile range) and comparisons made with Mann-Whitney rank rum test.

Variable	NODM	Control	Reference Range	*p*-Value
Number	14	14		
Glucose (mg/dL)	277.5 (311.8)	94.5 (18.7)	70–138	0.01
Fructosamine (µmol/L)	519.5 (175.5)	258.5 (65.5)	136–350	<0.001
Triglyceride (mg/dL)	408.5 (792.8)	178.0 (243.3)	29–291	0.04
Cholesterol (mg/dL)	510.0 (276.8)	249.0 (134.3)	92–324	<0.001

NODM, naturally occurring diabetes mellitus; *n*, number.

**Table 3:** Fungal species colonized from fecal cultures in dogs with naturally occurring diabetes mellitus and non-diabetic healthy controls.

Fungal Species	NODM	Control
*Candida glabrata*	4	0
*Candida albicans*	1	0
*Alternaria alternata*	1	1
*Curvularia lunata*	1	1
*Curvularia pallascens*	1	0
*Crysosporium shanxiense*	0	1
*Rhodotorula mucilaginosa*	1	1
*Aspergillus versicolor*	1	0
*Aspergillus nidulans*	1	0

NODM, naturally occurring diabetes mellitus.

**Table 4:** Comparative descriptive and clinicopathologic features of dogs with and without fungal colonization from fecal cultures.

Variable	Fungi-Positive	Fungi-Negative	*p*-Value
Number	16	12	
Age (years) ^a^	9.5 (2.5)	9.4 (1.8)	0.94 ^c^
Weight (kgs) ^b^	8.8 (15.7)	9.3 (39.6)	0.69 ^d^
Sex (male/female)	6, 10	10, 2	0.02 ^e^
Glucose (mg/dL) ^b^	157.0 (241.8)	97.0 (120.5)	0.28 ^d^
Fructosamine (µmol/L) ^b^	412.5 (307.5)	287.0 (231)	0.30 ^d^
Triglyceride (mg/dL) ^b^	267.5 (725.3)	198.5 (493.3)	0.80 ^d^
Cholesterol (mg/dL) ^b^	372.0 (367)	340.5 (256)	0.76 ^d^

kgs, kilograms, ^a^ Data presented as mean (standard deviation), ^b^ Data presented as median (interquartile range), ^c^ Two-tailed Student’s *t*-test, ^d^ Mann-Whitney rank sum test, ^e^ Fisher’s exact test.

**Table 5:** Comparative descriptive and clinicopathologic features of diabetic and non-diabetic control dogs with and without *Candida* spp. colonization from fecal cultures.

Variable	*Candida* spp.-Positive	*Candida* spp.-Negative	*p*-Value
Number	5	23	
Age (years) ^a^	10.6 (1.0)	9.2 (2.3)	0.19 ^c^
Weight (kgs) ^b^	23.4 (25.2)	8.8 (11.6)	0.47 ^d^
Sex (male/female)	3, 2	13, 10	1.00 ^e^
Glucose (mg/dL) ^b^	348.0 (226.5)	100 (123.0)	0.01 ^d^
Fructosamine (µmol/L) ^b^	594.0 (67.0)	279.0 (190.0)	0.002 ^d^
Triglyceride (mg/dL) ^b^	513.0 (684.0)	179.0 (487.0)	0.07 ^d^
Cholesterol (mg/dL) ^b^	608.0 (475.5)	301.0 (205.0)	0.01 ^d^

kgs, kilograms, ^a^ Data presented as mean (standard deviation), ^b^ Data presented as median (interquartile range), ^c^ Two-tailed Student’s t-test, ^d^ Mann-Whitney rank sum test, ^e^ Fisher’s exact test.

**Table 6:** Comparative descriptive and clinicopathologic features of dogs with naturally occurring diabetes mellitus with and without *Candida* spp. colonization from fecal cultures.

Variable	*Candida* spp.-Positive	*Candida* spp.-Negative	*p*-Value
Number	5	9	
Age (years) ^a^	10.6 (1.0)	8.8 (2.4)	0.13 ^c^
Weight (kgs) ^b^	23.4 (25.2)	8.4 (6.7)	0.29 ^d^
Sex (male/female)	3, 2	5, 4	1.00 ^e^
Glucose (mg/dL) ^b^	372.2 (114.6)	271.6 (216.5)	0.36 ^c^
Fructosamine (µmol/L) ^a^	588.8 (34.4)	428.7 (141.0)	0.03 ^c^
Triglyceride (mg/dL) ^b^	513.0 (684.0)	211.0 (807.5)	0.42 ^d^
Cholesterol (mg/dL) ^a^	672.2 (309.2)	479.7 (194.7)	0.17 ^c^

kgs, kilograms, ^a^ Data presented as mean (standard deviation), ^b^ Data presented as median (interquartile range), ^c^ Two-tailed Student’s t-test, ^d^ Mann-Whitney rank sum test, ^e^ Fisher’s exact test.

## Data Availability

The data presented in this study are openly available. Canine Diabetes Mellitus *Candida* spp. Available online: http://www.kaggle.com/jaredjaffey/canine-diabetes-mellitus-candida-spp?rvi=1 (accessed on 10 October 2022).

## Supplementary Materials

The following supporting information can be downloaded at: https://www.mdpi.com/article/10.3390/vetsci9100567/s1. Diet information for dogs with naturally occurring diabetes mellitus and non-diabetic controls.
