# Supplementary material for: Evaluation of Candida spp. and Other Fungi in Feces from Dogs with Naturally Occurring Diabetes Mellitus
Source: Vet Sci. 2022 Oct 16;9(10):567. doi: 10.3390/vetsci9100567 (PMC9609726; doi:10.3390/vetsci9100567)
Supplement: Supplementary file 1 [file vetsci-09-00567-s001.zip › vetsci-1956551-supplementary.pdf]

Supplemental Material

Dogs with naturally occurring diabetes mellitus were offered the following diets Hill's W/D kibble (4) [Hill's Pet Nutrition Inc., Topeka, KS, USA] Hill's Science Diet Adult Light with chicken kibble (1), Hill's W/D kibble and can (1), Hill's I/D kibble (1), Hill's Science Diet kibble and can (1), Royal Canin Glycobalance kibble and can (1) [Royal Canin SAS, Aimargues, France], Royal Canin Glycobalance (1), Nutro Salmon kibble (1) [Mars Inc, McLean, Virginia, USA], Fresh Pet (1) [Freshpet, Secaucus, NJ, USA, Honest Kitchen Whole Dehydrated Grain Chicken and Oat Recipe (1) [The Honest Kitchen, San Diego, CA, USA], and Dr. Harvey's (1) [Dr. Harvey's, Atlantic Highlands, NJ, USA].

Healthy control dogs were offered the following diets Royal Canin Adult Small kibble (3), Royal Canin Adult Medium kibble (1), Hill's Science Diet Mature (1), Blue Buffalo Chicken kibble (1) [Blue Buffalo Co., Ltd, Wilton, CT, USA], Grandma Mae's Grain free kibble (1) [Grandma Mae's Country Naturals, Manhattan, NY, USA], Pedigree Small Steak and Vegetable kibble (1) [Mars Inc.], Hill's Metabolic kibble (1), Hill's Science Diet Adult Small kibble (1), Hill's Science Diet Adult Large kibble (1), Rachael Ray Nutrish Chicken and Veggies kibble (1) [The J.M. Smucker Company, Orrville, OH, USA], Nutro Natural Choice Adult Chicken and Brown Rice kibble (1), Purina Beneful Healthy Weight kibble (1) [Nestle Purina PetCare Company, St. Louis, MO, USA].
